# Learning from work-from-home issues during the COVID-19 pandemic: Balance speaks louder than words

**DOI:** 10.1371/journal.pone.0261969

**Published:** 2022-01-13

**Authors:** Amanda M. Y. Chu, Thomas W. C. Chan, Mike K. P. So

**Affiliations:** 1 Department of Social Sciences, The Education University of Hong Kong, Tai Po, Hong Kong; 2 Department of Information Systems, Business Statistics and Operations Management, The Hong Kong University of Science and Technology, Clear Water Bay, Hong Kong; Shahrood University of Medical Sciences, ISLAMIC REPUBLIC OF IRAN

## Abstract

During the 2019 novel coronavirus disease (COVID-19) pandemic, many employees have switched to working from home. Despite the findings of previous research that working from home can improve productivity, the scale, nature, and purpose of those studies are not the same as in the current situation with the COVID-19 pandemic. We studied the effects that three stress relievers of the work-from-home environment–company support, supervisor’s trust in the subordinate, and work-life balance–had on employees’ psychological well-being (stress and happiness), which in turn influenced productivity and engagement in non-work-related activities during working hours. In order to collect honest responses on sensitive questions or negative forms of behavior including stress and non-work-related activities, we adopted the randomized response technique in the survey design to minimize response bias. We collected a total of 500 valid responses and analyzed the results with structural equation modelling. We found that among the three stress relievers, work-life balance was the only significant construct that affected psychological well-being. Stress when working from home promoted non-work-related activities during working hours, whereas happiness improved productivity. Interestingly, non-work-related activities had no significant effect on productivity. The research findings provide evidence that management’s maintenance of a healthy work-life balance for colleagues when they are working from home is important for supporting their psychosocial well-being and in turn upholding their work productivity.

## Introduction

### COVID-19 leads to working from home

Before the 2019 novel coronavirus disease (COVID-19) outbreak, most companies had not adopted the work-from-home (or working from home, WFH) approach. Employees needed to go to their offices on every working day. During the COVID-19 pandemic, individuals have been and are continuing to be advised to maintain social distancing to minimize the chance of infection [1]. To control the crisis, some countries and cities even need to institute lockdown measures to restrict the activities of their citizens [2]. However, under social distancing and lockdown policies, many employees are not able to go to their offices as usual. To maintain business operations, a majority of companies have responded improvisationally by introducing new WFH arrangements, although most of them have had little experience with such arrangements [3, 4]. Because WFH can reduce infection rates and is accompanied by the low economic costs of confinement [5], it should be a suitable measure for facing the COVID-19 challenge. However, not everyone is happy with working from home or is able to carry it out [6].

### Consequences of working from home

The WFH arrangements during the COVID-19 pandemic may have an impact on employees’ psychological well-being and, by extension, on their work performance. Because many employees have been forced to make WFH arrangements as a result of social distancing or lockdown policies during the COVID-19 pandemic, their WFH experiences may differ from those of employees in earlier studies, who were voluntarily working from home for a variety of reasons [4, 7, 8].

Indeed, the forced home confinement during lockdowns to control COVID-19 might affect individuals’ psychological well-being, including increasing their chances of disturbed sleep and insomnia because of the stressful situation and lack of positive stimuli [9]. Previous studies have confirmed the association between lockdown and negative psychological outcomes [10], such as higher stress levels [11]. However, the impact of WFH on workers’ psychological well-beings is not yet known. Being forced to engage in WFH but also unprepared for it may cause added stress on employees. On the positive side, remote employees have a high control of their working schedule and are able to work flexibly, which may have a positive impact on their job satisfaction [7]. They can adjust their working time so that they can fulfill other demands in their life, including family matters. A study [12] revealed that job flexibility could reduce work-to-home conflicts (conflicts caused by work issues interrupting home issues), and those reduced conflicts may help employees lower the distress of not fulfilling their family responsibilities.

Previous research has also suggested that positive psychological well-being is important for maintaining productivity in the workplace [13] although relatively little research has been done to study negative psychological well-being on employees’ job performance, especially during the WFH period. In addition, giving employees autonomy at home, along with controlling their boundaries, such as whether they conduct non-work-related activities during working hours, may be a great concern for employers [14]. According to the stress mindset theory, stress can be either enhance or debilitate one’s productivity [15] and growing evidence has shown that mindset shapes one’s stress response [16]. If employees hold the mindset that stress is debilitating, they will tend to focus on negative information from stressors, and that in turn will reinforce their negative beliefs and cause them to take action to avoid the stressors. In contrast, if employees hold the mindset that stress is enhancing, they will focus on positive information about stressors and will face their stresses and cope well with them [17]. By applying the stress mindset theory, we believe that when employees face stress, some can cope with it and maintain their focus on their work tasks while others may move on to other tasks to avoid the stress, instead of focusing on their work tasks. Those other tasks could be non-work-related activities, such as playing sports, shopping, and handling family matters. However, little empirical research has been conducted in these areas because they involve sensitive questions, such as whether the respondent is feeling stressed, and whether the respondent is conducting non-work-related activities during working hours [18]. Respondents are less willing to provide honest responses when they are asked such sensitive questions directly, and that dishonesty leads to response bias [19]. Therefore, we adopted the modified randomized response technique (RRT) to collect data on stress and non-work-related activities during working hours.

This research sought to investigate how the WFH environment affects individuals’ psychological well-being, and in turn how WFH impacts their work productivity and the frequency with which they conduct non-work-related activities during working hours when they are working from home.

## Materials and methods

### Methodology

#### Participants

A purposeful sample of 500 full-time employees in Hong Kong who experienced WFH for the first time during the COVID-19 pandemic was recruited online. The survey took place in early September 2020, which was near the end of the second period of growth in the number of confirmed COVID-19 cases in Hong Kong [20]. Table 1 shows a summary of the respondents’ demographic data. Such a diversity of participants reduces potential bias caused by the influence of socioeconomic backgrounds.

**Table 1:** Demographic characteristic of respondents.

Demographics	Number of respondents	% of respondents
**Gender**		
Male	212	42.4
Female	288	57.6
**Age group (in years)**		
18–24	52	10.4
25–34	175	35
35–44	115	23
45–54	126	25.2
55–64	31	6.2
65 or above	1	0.2
**Educational level**		
Non-bachelor’s degree or below	102	20.4
Bachelor’s degree or above	398	79.6
**Industry sector**		
Manufacturing	9	1.8
Wholesale and Retail	28	5.6
Import/Export Trade	16	3.2
Accommodations and Food Services	4	0.8
Education and Health Services	107	21.4
Real Estate and Business Services	24	4.8
Construction	19	3.8
Finance and Insurance	130	26
Transportation, Information and Communications	48	9.6
Social and Personal Services	44	8.8
Others	71	14.2
**Company size (number of employees)**		
1–49	104	20.8
50–99	80	16
100–199	63	12.6
200–499	46	9.2
500 employee or above	207	41.4
**Job position**		
Manager grade or above	138	27.6
Non-manager grade or below	362	72.4
	**500**	**100%**

#### Survey design

We identified our target respondents through personal networks and referrals, and then contacted them via emails and informed them of the study’s rationale. After confirming that the individuals were indeed our target respondents, we invited them to complete our self-administrated online questionnaire. All respondents were informed of the following in the first page of the online questionnaire: (1) the researcher’s name, affiliation, and contact details; (2) the topic and the aim of the study; and (3) the assurance that information about participation was anonymous and would be gathered on a voluntary basis. We obtained the respondents’ consent by asking them to click a button on the screen before starting the questionnaire. The study was conducted according to the prevailing guidelines on ethics in research, and it was approved by the Human Research Ethics Committee of The Education University of Hong Kong (reference number 2019-2020-0104).

#### Sensitive questions and confidentiality

To ensure full confidentiality of the participants’ responses, we made the survey anonymous, and applied the RRT for the sensitive questions about stress and non-work-related activities during working hours. We followed the guidance of Chong et al. (2019) and Chu et al. (2020) [18, 21] by implementing the RRT and constructing a covariance matrix for the responses. For details of the RRT procedure and application of RRT, readers may refer to Chong et al. (2019) and Chu et al. (2020) [18, 21].

To ensure that the respondents understood the purpose of using the RRT to further protect their privacy and clearly understood how to answer the RRT questions, we also included a brief introduction to the RRT procedures before we asked the RRT questions.

#### Measures

All items in the survey were measured on a seven-point Likert scale. Unless otherwise specified, we provided seven options for each item, ranging from 1 (strongly disagree) to 7 (strongly agree), and we asked each respondent to pick the option that best described the situation.

### Constructs and items

To build the research model, we constructed our survey questions on the basis of seven constructs, with each construct consisting of two to three items. A complete list of items is available in the S1 Table.

#### Company support

Communication with colleagues and access to technical support are important for enabling a smooth transition to WFH [22]. Following the work of Sull et al. (2020) [22], we developed three items to measure company support. A high score indicated strong support from the company for employees who were working from home.

#### Supervisor trust

When employees work from home, they have little opportunity to meet with their supervisors [23]. In the absence of supervisors and employees working face-to-face, supervisors’ trust in their subordinates is an important contribution to successful WFH [24]. We used three items to measure supervisor trust, with a high score indicating a high level of supervisors’ trust in their employees during WFH.

#### Work-life balance

A favorable environment and a healthy balance between working time and personal time could be an advantageous result of WFH [25]. With reference to Chaiprasit and Santidhirakul (2011) [26], we developed three items to measure work-life balance during WFH, with a high score indicating a good work-life balance.

#### Stress

On the basis of the existing literature, we developed three items to measure employees’ level of stress: sleep quality [27], loss of energy [28], and depressed mood [29]. A high score indicated a high level of stress during WFH.

#### Happiness

For the current study, we modified the three items relating to happiness that were developed by Chaiprasit and Santidhirakul (2011) [26]. The original items were in a five-point Likert scale, but we converted them into a seven-point Likert scale for measurement consistency in our study. A high score indicated a high level of happiness during WFH.

#### Non-work-related activities

During WFH, family issues and entertainment activities can distract employees from their work [30]. Following Ford et al. (2020) and Javed et al. (2019) [31, 32], we developed two items referring to these two possible distractions to measure the respondents’ non-work-related activities and we used a seven-point scale, ranging from 1 (never) to 7 (very many times), to quantify the respondents’ engagement in non-work-related activities [33]. A high score indicated a high frequency of conducting non-work-related activities during working hours when working from home.

#### Work productivity

We adopted the top three factors from the Endicott Work Productivity Scale [34] as items for measuring work productivity. The items were originally in a five-point scale, ranging from 1 (“never”) to 5 (“almost always”), but we modified the wording to adapt the scale to our context on WFH and our seven-point Likert scale approach. A high score indicated a high level of perceived productivity during WFH.

### Research model and hypotheses

#### WFH environment and psychological well-being

*Employees have had no choice but to work from home when their companies* or government policies have required it in response to the COVID-19 outbreak. For WFH to be successful, company support is necessary in three areas. First, some employees have insufficient equipment for WFH, and some may lack sufficient knowledge of the use of telecommunication technology [35]. Companies need to support their employees by providing them with the necessary equipment [36] and training them in the use of new technology [37]. Second, to avoid any impact of WFH on employees’ home time, companies have to set clear guidelines for distinguishing between work time and home time [38]. Third, companies have to decide when to start WFH and when to resume the normal working mode, and then they have to give their employees sufficient notice about the need to switch modes. We expected that company support during WFH would enhance job happiness [39] and would moderate the stresses from work and family. Therefore, we developed the following hypotheses:

**Hypothesis 1a**: Company support will negatively affect employees’ stress when they are working from home during the COVID-19 pandemic.
**Hypothesis 1b**: Company support will positively affect employees’ happiness when they are working from home during the COVID-19 pandemic.

As we have already noted, employers and employees do not see each other face-to-face in the WFH working environment. Thus, on one hand, employees have to show their employers that they are self-disciplined in completing their tasks on time and maintaining the expected quality of work [40] and, on the other hand, employers have to trust their employees that they have already tried their best in working on their assigned tasks [41]. In fact, some previous literature has mentioned that trust is the most critical factor in making WFH a success [42]. Therefore, we expected that supervisors’ trust in their subordinates would be important in maintaining employees’ happiness and reducing their stress on work [43]. Correspondingly, we developed the following hypotheses:

**Hypothesis 2a**: Supervisor trust will be negatively related to employees’ stress level when the employees are working from home during the COVID-19 pandemic.
**Hypothesis 2b**: Supervisor trust will be positively related to employees’ happiness when the employees are working from home during the COVID-19 pandemic.

A previous study of managers and fitness trainers discovered that loss of work-life balance could potentially boost the level of work-related stress because the workers spent extra time on work and did not have sufficient time for other life matters [44]. The association between a poor work-life balance and perceived job stress, which is caused by conflict between one’s job and other life activities, was further confirmed in a previous study on Australian academics [45]. The researchers explained that difficulty in maintaining work-life balance caused employees to feel additional stress. Moreover, research by Haar et al. (2014) [46] revealed that work-life balance was negatively related to depression across seven cultures in Asia, Europe, and Oceania, whereas work-life balance was positively associated with job and life satisfaction. Another study on healthcare employees also discovered a positive relationship between work-life balance and job satisfaction [47]. In addition, Fisher (2003) [44] found that having a good work-life balance could minimize the interference between employees’ work life and their personal life, thus allowing them to maintain their job engagement and family involvement at the same time, and fostering greater happiness in their work. Thus, we formulated the following two hypotheses:

**Hypothesis 3a**: Work-life balance will be negatively related to employees’ stress level when they are working from home during the COVID-19 pandemic.
**Hypothesis 3b**: Work-life balance will be positively related to employees’ level of happiness when they are working from home during the COVID-19 pandemic.

#### Psychological well-being, non-work-related activities, and productivity

Previous studies have revealed the causal relationship that increased stress leads to a reduction in employees’ productivity [48–50]. Indeed, chronic stress can have several negative effects on employees, including insomnia, concentration difficulty, and increased risk of depression, all of which are likely to reduce productivity.

Some employees may choose to conduct non-work-related activities (e.g., non-work-related computing) while at work [33]. In our context, non-work-related activities are not referring to necessary activities such as going to the washroom or having a short break. We are considering situations in which an employee chooses to conduct non-work-related activities during work hours even if he or she could do those activities later. The reasons for conducting non-work-related activities during work hours are varied. Some studies have suggested that non-work-related activities can be caused by resistance and lack of management [51, 52]. If an employee has a negative impression of the company or of management, that worker will have a low level of working engagement. In other words, a stressful working environment or management style can generate negative feelings in employees, and those negative feelings may motivate them to do something unrelated to their work during work hours. Accordingly, we formulated Hypotheses 4a and 4b as follows:

**Hypothesis 4a**: Employees’ stress level will be negatively related to their work productivity when they are working from home during the COVID-19 pandemic.
**Hypothesis 4b**: Employees’ stress level will be positively related to employees’ participation in non-work-related activities during working hours when they are working from home during the COVID-19 pandemic.

In contrast, happiness can have a positive impact on employees’ productivity. Under a classic piece rate setting, happier individuals have greater productivity than less happy individuals do, no matter whether the happiness derives from long-term or short-term events [53]. If employees think that they can achieve happiness by performing better at work, they will work harder for that reinforcement [54]. Therefore, the following hypothesis was also included:

**Hypothesis 5**: Employees’ happiness will be positively related to their work productivity when they are working from home during the COVID-19 pandemic.

Moreover, employees may have difficulty in concentrating on their work when they are working from home because of the lack of an organizational climate and in response to interruptions from family members [55]. In particular, employees who have children need to shoulder extra child care duties because of school closures [56, 57]. At the same time, a feeling of insecurity because of rising numbers of COVID-19 cases also can distract employees [10], perhaps promoting them to conduct non-work-related activities during working hours at home to drive themselves out from the feeling of insecurity. Two major types of non-work-related activities are (1) activities fulfilling some demand in one’s life, such as caring for children, doing housework, or other activities that the person cannot escape when working from home; and (2) entertainment activities, such as playing video games and sports during working hours [31, 32]. Some previous research has suggested that conducting non-work-related activities at work, such as using the Internet for personal purposes in the workplace, can affect job performance [52, 58]. Hence, the final hypothesis we postulated was as follows:

**Hypothesis 6**: Employees’ participation in non-work-related activities during working hours will be negatively related to their work productivity when they are working from home during the COVID-19 pandemic.

### Statistical analysis

We tested our hypotheses using structural equation modeling (SEM) in AMOS statistical software. The main purpose of using SEM in our analysis was to test the hypotheses about the constructs that we determined from the observed items we collected from the respondents [59].

To ensure that our model had a consistent construction, we analyzed the convergent validity and discriminant validity of the constructs by considering their Cronbach’s alpha values, average variance extracted (AVE) values, and square root of AVE values, on the respective constructs and the item loadings. Cronbach’s alpha measures the internal consistency of constructs [60]. The average variance extracted provides the average of variation explained by a construct [61].

Moreover, we assessed the model fit using (1) absolute fit indexes, including the goodness-of-fit index (GFI) and the root mean square error of approximation (RMSEA), and (2) incremental fit indexes, including the comparative fit index (CFI) and the normed fit index (NFI) [62].

After confirming that the model was consistent and had a good fit, we examined the model by SEM. We then calculated the significance of each path using a two-tailed *t*-test to test the cause and effect relationships among the constructs.

## Results

### Model consistency

We list the summary statistics, including the mean and standard deviation of each item, the item loadings, and the Cronbach’s alpha of each construct in Table 2. The correlations between constructs, average variances extracted (AVEs), and the square roots of the AVEs are listed in Table 3. The Cronbach’s alpha of each construct was above the benchmark value of acceptable reliability 0.7 [63], thus suggesting a good internal consistency of each construct. In order to ensure that each item represented its construct, each item needed to have a loading larger than 0.4 [64, 65]. All of the item loadings in our research exceeded 0.4, and the AVE value for each construct was larger than 0.5 (except one, which was 0.5), thus demonstrating that the items satisfied the requirements for convergent validity [66, 67]. In addition, the square root of the AVE of each construct was larger than its correlations with all of the other constructs [67] meaning that the discriminant validity was at an acceptable level.

**Table 2:** Summary statistics of items and factor loadings.

Item (Construct)	Mean	Standard deviation	Item loading	Cronbach’s alpha
**Company support (COM)**				0.859
COM1	5.122	1.601	0.856	
COM2	5.362	1.576	0.899	
COM3	4.712	1.604	0.712	
**Supervisor trust (SUT)**				0.851
SUT1	4.658	1.592	0.848	
SUT2	4.592	1.613	0.840	
SUT3	4.846	1.600	0.744	
**Work-life balance (WLB)**				0.840
WLB1	4.564	1.554	0.743	
WLB2	4.776	1.623	0.882	
WLB3	5.024	1.463	0.789	
**Stress (STR)**				0.736
DEP1	3.308	1.758	0.448	
DEP2	3.256	1.748	0.714	
DEP3	3.284	1.672	0.889	
**Happiness (HAP)**				0.894
HAP1	4.924	1.577	0.813	
HAP2	4.468	1.525	0.926	
HAP3	4.014	1.527	0.854	
**Non-work-related activities (NWA)**				0.704
NWA1	3.708	1.551	0.566	
NWA2	2.968	2.027	0.960	
**Work productivity (WKP)**				0.923
WKP1	3.768	1.827	0.907	
WKP2	3.788	1.793	0.945	
WKP3	3.736	1.763	0.837	

**Table 3:** Correlations of the constructs.

	AVE	COM	SUT	WLB	STR	HAP	NWA	WKP
**COM**	0.682	0.826						
**SUT**	0.659	0.336	0.812					
**WLB**	0.651	0.167	0.172	0.807				
**STR**	0.500	0.013	-0.096	-0.224	0.707			
**HAP**	0.749	0.194	0.128	0.759	-0.164	0.865		
**NWA**	0.621	0.008	-0.060	-0.140	0.626	-0.103	0.788	
**WKP**	0.806	0.080	0.064	0.341	-0.177	0.436	-0.119	0.898

The diagonal elements represent the square root of the average variance extracted (AVE). COM is the company support, SUT is the supervisor trust, WLB is the work-life balance, STR is stress, and HAP is happiness, NWA is non-work-related activities, and WKP is the participant’s work productivity.

### Model goodness of fit

The cut-off criteria of a good model fit are: RMSEA < 0.06, and GFI, CFI, and NFI ≥ 0.9 [68–71]. In this case, the study’s model demonstrated a satisfactory fit (RMSEA = 0.061; CFI = 0.947; GFI = 0.919; NFI = 0.922).

### Testing of hypotheses

**Fig 1:**
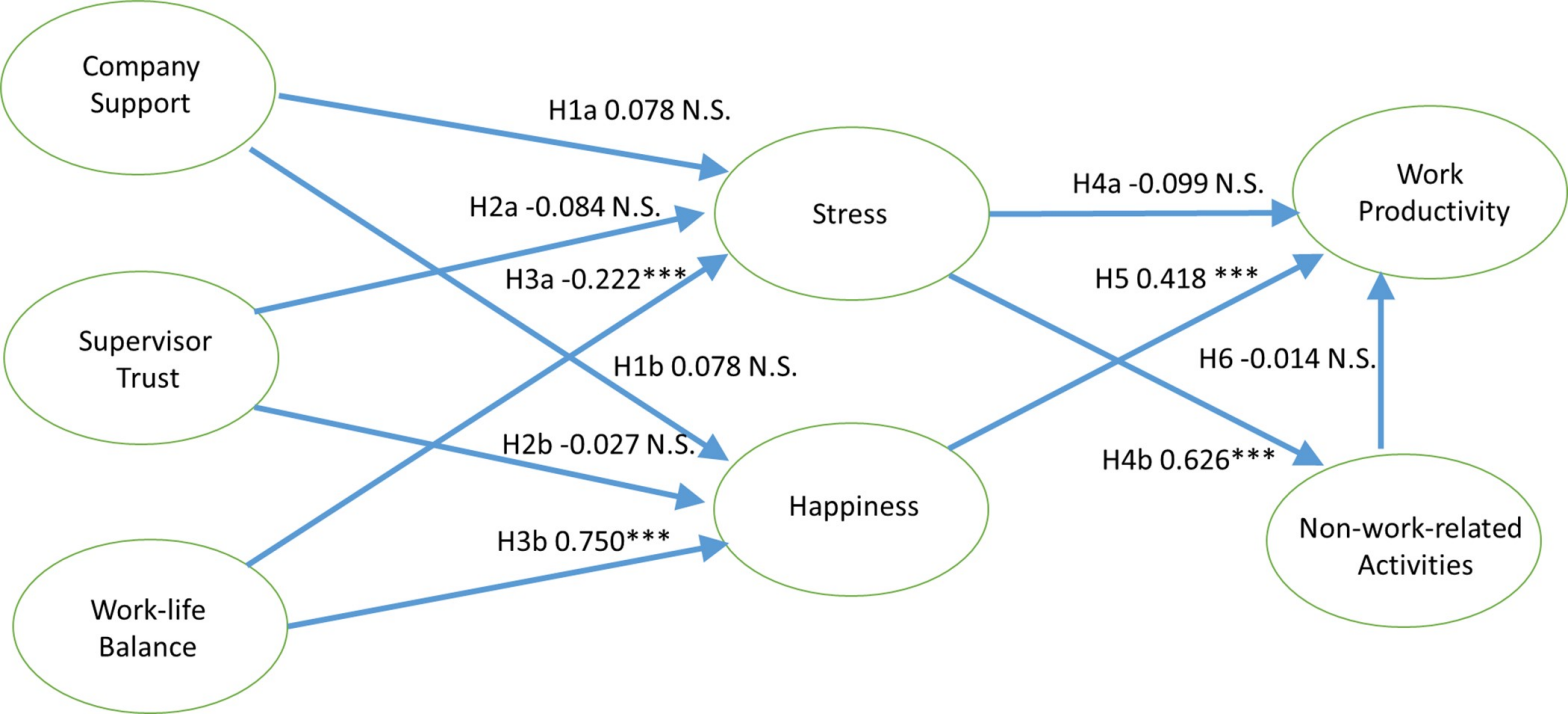
Results of the research model testing. N.S. represents not significant. *** indicates a *p*-value less than 0.01. The numbers to the right of the hypotheses’ numbers are the standardized path coefficients.

We report the standardized path coefficients and the significance of each of the hypotheses in Fig 1. Based on a significance level of 5%, four hypotheses were significant and six were not significant.

The research findings supported Hypotheses H3a, H3b, H4b, and H5. Hypothesis H3a was supported (*β* = -0.222, *p* < 0.001), indicating that work-life balance was negatively related to the employees’ stress level when those employees were working from home during the COVID-19 pandemic. Hypothesis H3b was also supported (*β* = 0.750, *p* < 0.001), indicating that employees’ work-life balance was positively related to their happiness when those employees were working from home during the COVID-19 pandemic. Hypothesis H4b was supported (*β* = 0.626, *p* < 0.001), indicating that employees’ stress level was positively related to the employees’ participation in non-work-related activities during working hours when those employees were working from home during the COVID-19 pandemic. Hypothesis H5 was supported (*β* = 0.418, *p* < 0.001), indicating that employees’ happiness had a positive effect in promoting their work productivity.

## Discussion

The COVID-19 pandemic has forced many employees who were accustomed to working in the office and did not have previous WFH experience to do their work from home during part of the pandemic, because of social distancing or lockdown policies. In this research, we sought to investigate the effects that switching to WFH in response to the COVID-19 pandemic had on employees’ psychological well-being and, by extension, on their work productivity. We applied the stress mindset theory to study the relationships between three stress relievers (company support, supervisor trust, and work-life balance) on the positive and negative sides of employees’ psychological well-being (happiness versus stress), which in turn affected their job performance (productivity and non-work-related activities during working hours) when they were working from home during the COVID-19 pandemic. Interestingly, among the three stress relievers we studied, work-life balance is the only reliever that have influenced on the employees’ psychological well-being. At the same time, this reliever has a positive effect on one’s psychological well-being by promoting happiness and relieving stress. Our research findings also suggest that when employees feel happy in their WFH arrangements, their work productivity increases. Surprisingly, when the employees encountered stress in their WFH arrangements, they still maintained their work productivity, but at the same time, they participate more in non-work-related activities to relieve their stress. The good news is that their non-work-related activities did not affect their work productivity. Our study takes the lead in developing a research model that shapes the relationship between employees’ WFH environment and their psychological well-being and performance in relation to sudden and forced WFH during the COVID-19 pandemic. As a methodological contribution, our study adopted the modified randomized response technique to ask the sensitive questions involved in the study, including queries about the employees’ negative psychological well-being status and their engagement in non-work-related activities. We provided extra protection to their privacy by using this survey method, so as to encourage them to provide truthful responses when answering such sensitive questions. Management may wish to consider adopting the same methodology in an effort to collect honest responses when sensitive questions are involved in the workplace.

Regarding the effect of stress relievers on psychological well-being, we found that having a healthy work-life balance promotes happiness and also relieves stress. However, WFH does not imply an improvement in work-life balance, especially when the employees do not have a suitable environment to work. Employees should have a private workspace, which allows access to a strong and stable Internet connection, and has sufficient equipment to carry out their work at home. If employees encounter difficulties when they are working from home, management should provide the employees with flexible arrangements and alternative approaches to work. For example, if an employee does not have a comfortable environment to work, management may arrange a private space or room in the office for the employees given that a proper social distance is maintained.

As is the case in other fast-paced metropolises, Hong Kong has long followed the standard practice of employees working in a formal office environment and offering them no flexible working options [72]. During the pandemic, when the employees are allow to work from home, some companies have also set strict rules, such as requiring staff to stay at home during working hours or to answer calls from supervisors within three tones. However, a blurred boundary between work space and home space can make it difficult for employees to set a clear line of separation between their work and their home life [73]. Under a work-life balance working approach, it is assumed that employees can reserve enough time to handle non-work-related life issues and activities while managing their work tasks. Although some previous studies have suggested that non-work-related activities in the workplace affect work productivity [52, 58], our research findings did not support that argument in regard to WFH. In other words, performing non-work-related activities during work hours at home does not necessarily appear to impact work productivity. In fact, when employees are feeling burned-out, they could relieve stress via such non-work-related activities and hence maintain their work engagement. For example, at the time when use of the Internet was just emerging in the workplace, Internet recreation in the workplace was found to make employees more creative [74] and help employees to become accustomed to the new and advanced systems [75].

Therefore, management may wish to offer their employees a flexible working hour to help the employees to meet their needs when they are working from home [57]. Management could also encourage employees to set boundaries, as long as the committed working hours per week are achieved, thereby enabling them to secure the balance between their work and home life. Feeling happy, satisfied, and enthusiastic when working from home can help workers maintain a high level of productivity [76].

### Limitations and future research

The present study had certain limitations. First, the significance of the research findings is dependent on the reliability of self-reports. To minimize bias, in this study we attempted to collect the most representative responses, including through application of the RRT for sensitive questions and through use of an anonymous, web-based survey, as well as through the choice of highly diverse participants. A pretest and pilot test were also conducted before the actual survey, to ensure the quality of the study. Second, this study was based on 500 employees in Hong Kong, a group that certainly cannot represent the worldwide population. In addition, the working and living environments in Hong Kong may be significantly different from those in other regions or countries. Additionally research among more heterogeneous samples will be needed to test the research model.

## Conclusions

Although managers are trying their best to maintain their employees’ work productivity at the same level as that prior to the COVID-19 pandemic, it is also important for them to maintain a good balance for their employees between work and life and provide flexibility in their working time and arrangements. Our research findings suggest that a healthy balance between work and home life makes employees feel happier, and in turn has a significant effect on them maintaining a good level of work productivity when they are required to switch to WFH. Meanwhile, an imbalance between work and life would have a negative impact on employees’ psychological well-being, spurring them to carry out non-work-related activities during working hours. Interestingly, those non-work-related activities apparently do not influence WFH employees’ work productivity. We conclude that balance is the key to successful implementation of sudden and forced WFH during the COVID-19 pandemic and achieving a smooth transition from working at the office to working from home.

## Supporting information

S1 Table List of all items and measures. Suffixes with–S and–U indicate that the items are sensitive questions and are paired with unrelated questions.
(DOCX)
