# Supplementary material for: Learning from work-from-home issues during the COVID-19 pandemic: Balance speaks louder than words
Source: PLoS One. 2022 Jan 13;17(1):e0261969. doi: 10.1371/journal.pone.0261969 (PMC8758108; doi:10.1371/journal.pone.0261969)
Supplement: S1 Table — Suffixes with–S and–U indicate that the items are sensitive questions and are paired with unrelated questions. (DOCX) [file pone.0261969.s001.docx]

**S1 Table. List of all items and measures.** Suffixes with –S and –U indicate that the items are sensitive questions and are paired with unrelated questions.

| **Company support** | |
| --- | --- |
| COM1 | My company provides me with clear guidelines on working from home. |
| COM2 | My company keeps me well informed of the work from home arrangement. |
| COM3 | My company paid every effort (including technological support) to ensure that work from home runs smoothly. |
| **Supervisor trust** | |
| MUT1 | My supervisor does not pressure me much when I work from home. |
| MUT2 | My supervisor does not pressure me much when I am unable to meet deadlines set by them/the company when work from home. |
| MUT3 | My supervisor fully trusts my work performance when I work from home. |
| **Work-life balance** | |
| WLB1 | My home provides me with a good working environment that promotes both physical and mental well-being. |
| WLB2 | Working from home can provide me a better balance between personal and work life than when I work at the office. |
| WLB3 | Working from home makes it possible for me to manage time both for personal matters and work. |
| **Stress *(RRT is applied)*** | |
| DEP1-S | Compared with normal working periods, I get more depressed during work from home periods. |
| *DEP1-U* | *I think being a vegetarian can improve my health.* |
| DEP2-S | Compared with normal working periods, my sleep quality is bad during work from home periods. |
| *DEP2-U* | *I think vegetarianism can reduce global warming.* |
| DEP3-S | Compared with normal working periods, I have less energy during work from home periods. |
| *DEP3-U* | *I think recording personal daily expenses in detail is a wise idea.* |
| **Happiness** |  |
| HAP1 | I feel more joy at work when working from home than when working at the office. |
| HAP2 | I feel more satisfied with work when working from home than when working at the office. |
| HAP3 | I feel more enthusiastic at work when working from home than when working at the office. |
| **Non-work-related activities *(RRT is applied)*** | |
| NWA1-S | Handle family issues (e.g., home cleaning, reading with children) during office hours during work from home periods. |
| *NWA1-U* | *Make online purchase.* |
| NWA2-S | Indulge in entertainment activities (e.g., shopping, sports) during office hours during work from home periods. |
| *NWA2-U* | *Use Octopus card.* |
| **Work productivity** | |
| WKP1 | I notice my productivity is higher when working from home than when working at the office. |
| WKP2 | I notice my efficiency is higher when working from home than when working at the office. |
| WKP3 | I work quicker or take less time to complete tasks when working from home than when working at the office. |
